# Cyclic Peptide-Based Sirtuin Substrates

**DOI:** 10.3390/molecules24030424

**Published:** 2019-01-24

**Authors:** Di Chen, Lingling Yan, Weiping Zheng

**Affiliations:** School of Pharmacy, Jiangsu University, 301 Xuefu Road, Zhenjiang 212013, China; jsdxchendi@163.com (D.C.); 18796012926@163.com (L.Y.)

**Keywords:** sirtuin, deacylation, substrate, cyclic peptide

## Abstract

In the current study, four side chain-to-side chain cyclic peptides (three 5-mers and one 4-mer) harboring N^ε^-acetyl-lysine or N^ε^-myristoyl-lysine were found to be in vitro substrates of the human SIRT1/2/3-catalyzed deacylation with good substrate activities, as judged by the *k*_cat_/*K*_M_ ratios.

## 1. Introduction

**Figure 1:**
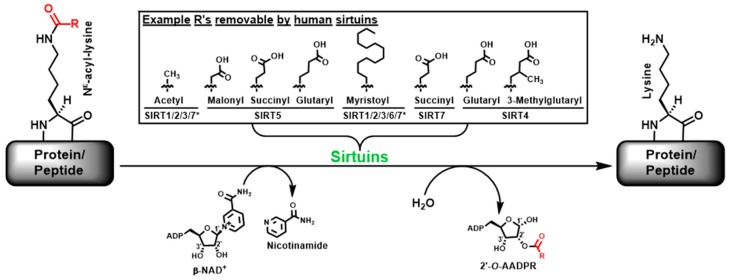
The sirtuin-catalyzed β-NAD^+^-dependent deacylation of protein-based or peptide-based N^ε^-acyl-lysine substrates. Example R groups of R-C(=O)- removable by different human sirtuins are shown in the box. The two asterisks in the box denote that the SIRT7-catalyzed deacetylation and demyristoylation become appreciable only in the presence of dsDNA, rRNA, or tRNA.

Sirtuins refer to a family of intracellular enzymes able to catalyze the β-nicotinamide adenine dinucleotide (β-NAD^+^)-dependent N^ε^-acyl-lysine deacylation on histone and non-histone proteins in organisms from all three evolutionary kingdoms of life (i.e., bacteria, archaea, and eukarya) [1,2,3,4,5,6,7,8]. Figure 1 depicts this enzymatic reaction in which the N^ε^-acyl-lysine substrate, β-NAD^+^, and water are converted to nicotinamide, deacylated product, and 2′-*O*-acyl-ADP ribose (2′-*O*-AADPR) [2,3,4,5,6,7]. The N^ε^-acyl specificity exhibited by the seven human sirtuins (i.e., SIRT1-7) is also shown in Figure 1 [2,3,4,5,6,7]. Specifically, while human SIRT1/2/3 are the three sirtuins that are able to proficiently catalyze the deacetylation of N^ε^-acetyl-lysine, they are also able to proficiently catalyze the demyristoylation of N^ε^-myristoyl-lysine; SIRT5 has strong N^ε^-malonyl/succinyl/glutaryl-lysine demalonylase/desuccinylase/deglutarylase activities; SIRT6 is a strong N^ε^-myristoyl-lysine demyristoylase; SIRT4 was very recently found to have strong N^ε^-glutaryl-lysine and N^ε^-3-methylglutaryl-lysine deacylase activities; and very recently, SIRT7 was found to be a N^ε^-succinyl-lysine desuccinylase. Moreover, SIRT7’s deacetylase and demyristoylase activities were found to be significantly enhanced by the presence of double-stranded DNA (dsDNA), ribosomal RNA (rRNA), and transfer RNA (tRNA).

**Figure 2:**
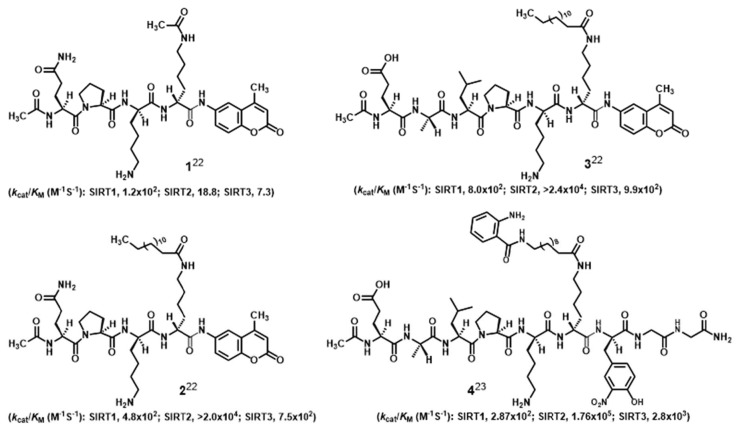
Notable examples of the currently existing in vitro SIRT1/2/3 substrates. Note: (i) the *k*_cat_/*K*_M_ values for the SIRT1/2/3-catalyzed deacylation of these substrates are shown in the parenthesis below each chemical structure. (ii) The sequence of peptides **1** and **2** was derived from tumor suppressor protein p53, and that of peptides **3** and **4** was derived from the tumor necrosis factor α (TNFα).

**Figure 3:**
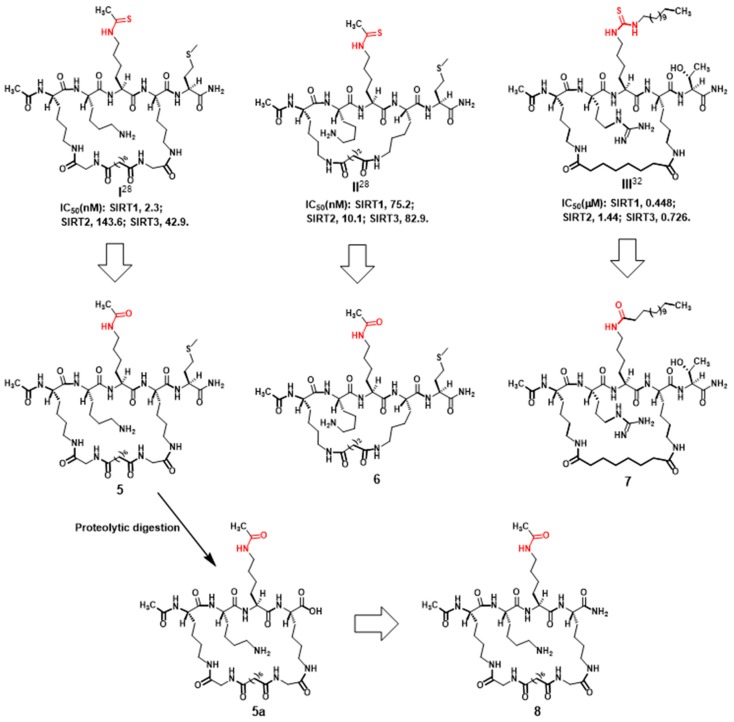
The design of compounds **5**–**8**.

The eukaryotic sirtuins including human sirtuins are found in nucleus, mitochondria, and cytosol [2,5,7,8]. In addition to histone proteins, the first identified eukaryotic sirtuin substrates, more and more non-histone substrates have also been found in the cellular compartments where sirtuins reside [2,5,7,8]. Therefore, it is not surprising that the sirtuin-catalyzed deacylation has been demonstrated to play an important regulatory role in multiple crucial cellular processes, e.g., transcription, metabolism, and DNA damage repair [2,5,9,10,11,12,13,14]. This enzymatic reaction has also been regarded as a novel therapeutic target for multiple human diseases such as cancer and the metabolic and neurodegenerative diseases [15,16,17,18]. Therefore, chemical modulators (inhibitors and activators) for the sirtuin-catalyzed deacylation have been actively pursued during the past few years [4,15,17,18,19], and quite a few in vitro sirtuin substrates have been developed and employed on in vitro screening platforms for such chemical modulators [20,21,22,23,24,25,26]. Given that the notable examples of the currently existing in vitro SIRT1/2/3 substrates **1**–**4**, as shown in Figure 2, are all linear peptide-based, we envisioned that cyclic peptides could be superior substrates because cyclic peptides tend to have superior target binding affinity [27]. As a proof-of-concept endeavor, in the current study we prepared and assessed the SIRT1/2/3 substrate activities of cyclic peptides **5**–**8** shown in Figure 3. We found that, as judged by *k*_cat_/*K*_M_ ratios, cyclic peptides **5**, **7**, and **8** were superior in vitro SIRT1 or SIRT3 substrates compared to the best linear hexapeptide-based in vitro SIRT1 or SIRT3 substrates reported in the current literature and shown in Figure 2.

## 2. Results and Discussion

### 2.1. Compound Design

It should be first noted that, in the current study the cyclic counterparts of the currently existing linear peptide-based SIRT1/2/3 substrates **1**–**4** were not pursued due to the need of an effort to determine the macrocycle bridging units that can be accommodated favorably at SIRT1/2/3 active sites; instead, we came up with the cyclic peptides **5**–**8** depicted in Figure 3 based on the following straightforward design.

Compounds **I** and **II** were found previously in our laboratory to be potent SIRT1/2/3 inhibitors [28]. While this strong inhibition could be derived from the use of the powerful catalytic mechanism-based SIRT1/2/3 inhibitory warhead N^ε^-thioacetyl-lysine [29,30] (the central residue in both compounds), it also suggested that the SIRT1/2/3 active sites were able to favorably accommodate the macrocycle bridging units in these two compounds which ought to be first recognized and processed by SIRT1/2/3 as substrates [29,30]. Therefore, we envisioned that simply replacing acetyl for thioacetyl in compounds **I** and **II** could afford robust SIRT1/2/3 substrates **5** and **6**.

Since SIRT1/2/3 were also recently found to exhibit robust demyristoylase activity [26,31], it seemed plausible to construct another robust SIRT1/2/3 substrate by simply replacing the acetyl in compounds **5** and **6** with myristoyl, however, this replacement may result in a sub-optimal positioning of the macrocycle bridging units of **5** and **6** at SIRT1/2/3 active sites. Therefore, we opted to use the macrocyle bridging unit of compound **III** in Figure 3 that was found previously in our laboratory to also be a strong SIRT1/2/3 inhibitor [32]. Again, this observed strong inhibition could have resulted from the use of the depicted thiourea-type catalytic mechanism-based sirtuin inhibitory warhead [18] (i.e., the central lysine-derivatized residue); it also suggested that the SIRT1/2/3 active sites were able to favorably accommodate the specific macrocycle bridging unit in compound **III** that also ought to be first recognized and processed by SIRT1/2/3 as substrate [33]. Therefore, the macrocycle bridging unit of compound **7** is the same as that of compound **III** while the thiourea-type warhead in **III** was replaced with N^ε^-myristoyl-lysine in **7**.

### 2.2. Compound Preparation

**Scheme 1:**
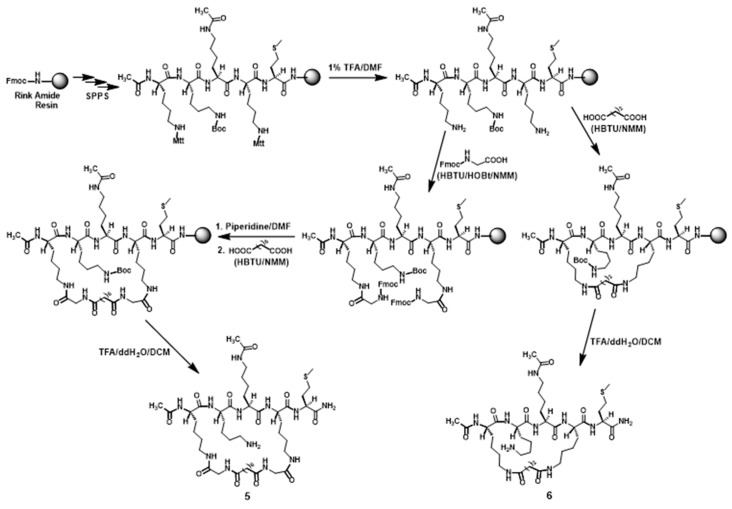
The synthetic scheme of compounds **5** and **6**.

Compounds **5** and **6** were synthesized according to Scheme 1. Following the assembly and the N-term acetylation of the linear pentapeptide composed of Met, Lys(Mtt), N^ε^-acetyl-Lys, Lys(Boc), and Lys(Mtt) on the Rink amide resin based on the N^α^-9-fluorenylmethoxycarbonyl (Fmoc) chemistry-based manual solid phase peptide synthesis (SPPS), the two side chain Mtt protecting groups were removed with 1% (*v*/*v*) trifluoroacetic acid (TFA)/*N*,*N*-dimethylformamide (DMF). For the synthesis of compound **5**, the resulting peptidyl-resin with two exposed free amino groups was then reacted with Fmoc-Gly-OH followed by (i) Fmoc removal with 20% (*v*/*v*) piperidine/DMF and (ii) the subsequent reaction between the newly exposed two free amino groups on resin with suberic acid (2 equivalents) in the presence of 2-(1H-benzotriazole-1-yl)-1,1,3,3-tetramethylaminium hexafluorophosphate (HBTU) (3.8 equivalents) and 0.4 M N-methylmorpholine (NMM)/DMF at room temperature for 1 h. For the synthesis of compound **6**, the peptidyl-resin following Mtt removal was directly reacted with a di-acid (succinic acid, 2 equivalents) in the presence of HBTU (3.8 equivalents) and 0.4 M NMM/DMF at room temperature for 1 h. The above-described peptidyl resin obtained from the reaction with a di-acid (suberic acid or succinic acid) was then treated with a TFA-containing cocktail, affording the crude **5** or **6,** which was purified with semi-preparative reversed-phase high performance liquid chromatography (RP-HPLC). The exact masses of the purified **5** and **6** were confirmed by high-resolution mass spectrometry (HRMS) analysis (Table 1). The purified **5** and **6** were each >95% pure based on a RP-HPLC analysis.

**Scheme 2:**
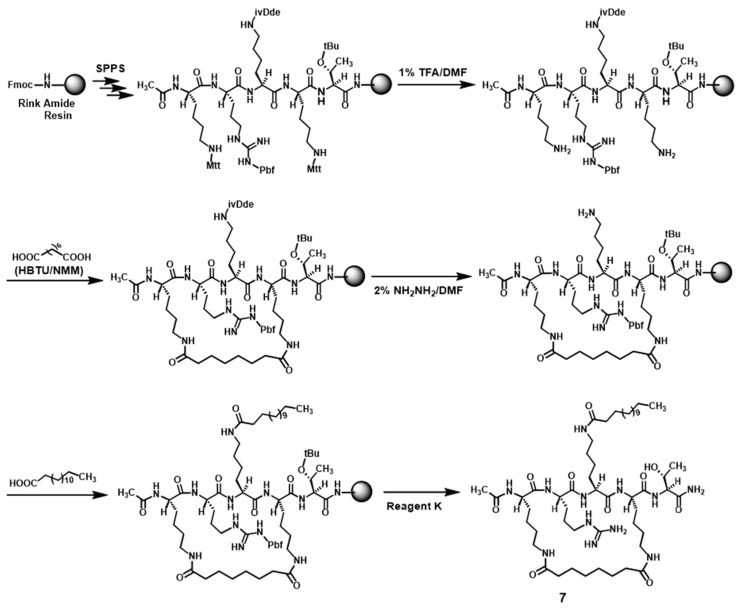
The synthetic scheme of compound **7**.

Scheme 2 depicts the synthesis of compound **7**. Following the assembly and the N-term acetylation of the linear pentapeptide composed of Thr(tBu), Lys(Mtt), Lys(ivDde), Arg(Pbf), and Lys(Mtt) on the Rink amide resin based on the Fmoc chemistry-based manual SPPS, the two side chain Mtt protecting groups were removed with 1% (*v*/*v*) TFA/DMF. The resulting peptidyl-resin with two exposed free amino groups was then reacted with suberic acid (2 equivalents) in the presence of HBTU (3.8 equivalents) and 0.4 M NMM/DMF at room temperature for 1 h. The ivDde protecting group on the obtained peptidyl-resin was then selectively removed with 2% (*v*/*v*) hydrazine/DMF, and the newly exposed free amino group on the resin was subsequently reacted with myristic acid. The crude **7** was then cleaved from the resin with Reagent K (a TFA-containing cocktail) and purified by semi-preparative RP-HPLC. The exact mass of the purified **7** was confirmed by HRMS analysis (Table 1). The purified **7** was >95% pure based on a RP-HPLC analysis.

**Scheme 3:**
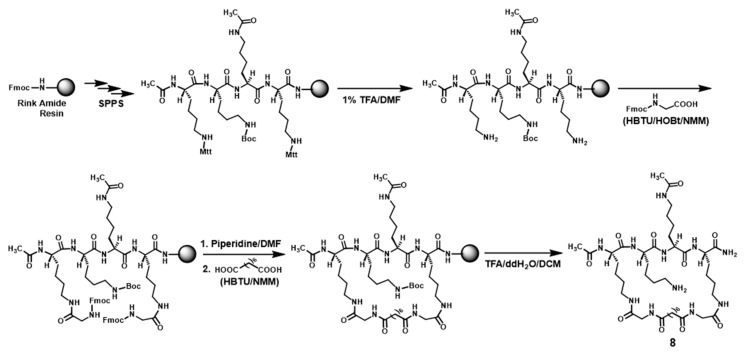
The synthetic scheme of compound **8**.

The 4-mer **8** was synthesized (see Scheme 3) in the same manner as the above-described synthesis of the 5-mer **5**. The crude **8** was also purified with semi-preparative RP-HPLC and the exact mass of the purified **8** was also confirmed by HRMS analysis (Table 1). The purified **8** was also >95% pure based on a RP-HPLC analysis.2.3. Compound Evaluation with In Vitro SIRT1/2/3 Substrate Activity Assay

The steady-state kinetic parameters (*k*_cat_ and *K*_M_) for the SIRT1/2/3 substrate activities of the purified **5**–**8** were determined with a HPLC-based sirtuin deacylation activity assay and are recorded in Table 2 and Table 3. As judged by *k*_cat_/*K*_M_ ratios, cyclic peptides **5**–**8** were all found to be excellent SIRT1 substrates, actually **5**, **7**, and **8** are all better SIRT1 substrates than the linear hexapeptide-based SIRT1 substrate **3** (depicted in Figure 2) reported in the current literature (*k*_cat_/*K*_M_ = 800 M^−1^ s^−1^) [22]. Cyclic peptides **5**–**8** were also all found to be better SIRT3 substrates than the linear hexapeptide-based SIRT3 substrate **3** reported in the current literature (*k*_cat_/*K*_M_ = 990 M^−1^·s^−1^) [22]. Even though cyclic peptides **5**–**8** were found to be ~17-68-fold weaker SIRT2 substrates than the best SIRT2 substrate (i.e., **4** depicted in Figure 2) reported in the current literature (*k*_cat_/*K*_M_ = 1.76 × 10^5^ M^−1^·s^−1^) [23], this literature SIRT2 substrate was based on a linear nonapeptide.

### 2.3. Compound Evaluation with Pronase Digestion Assay

**Figure 4:**
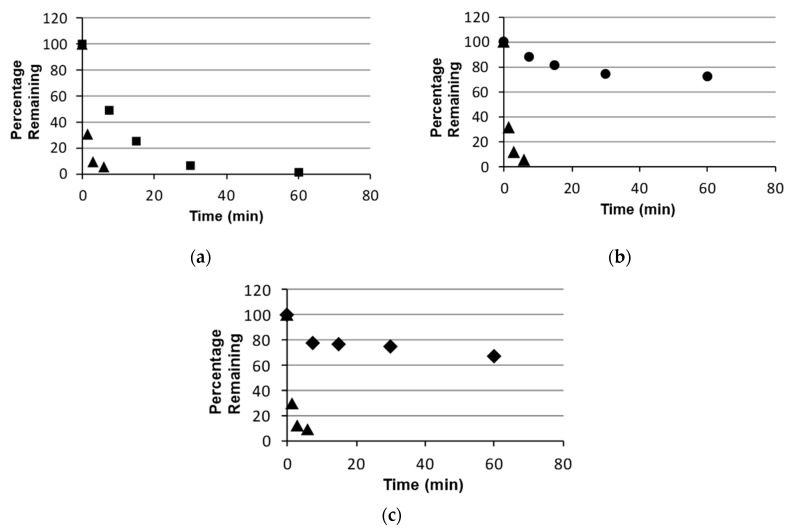
The pronase digestion profiles of compounds **5** (■), **6** (●), **7** (♦), and the linear pentapeptide H_2_N-HK-(N^ε^-acetyl-lysine)-LM-COOH (▲). Note: The linear pentapeptide H_2_N-HK-(N^ε^-acetyl-lysine)-LM-COOH has been used extensively in our laboratory as a control of the pronase digestion assay.

With compounds **5**–**7** in hand, we also performed a proteolysis experiment to assess the proteolytic stability of these three cyclic peptides. Pronase was employed in the experiment as the proteolytic enzyme preparation since pronase is composed of a variety of different types of proteases and peptidases, and thus, has a very broad substrate specificity [34]. As indicated in Figure 4, compounds **6** and **7** were found to be proteolytically much more stable than compound **5**, while these three cyclic peptides are all proteolytically much more stable than the linear pentapeptide H_2_N-HK-(N^ε^-acetyl-lysine)-LM-COOH also employed in the proteolysis experiment. Given the relatively lower proteolytic stability of **5**, we also set out to identify its stable proteolysis product(s) and found one such product via HRMS analysis, compound **5a** (depicted in Figure 2): HRMS (electrospray ionization) calculated for C_40_H_71_N_10_O_11_ ([M + H]^+^) 867.5298; found: 867.5295. Subsequently, we prepared compound **8**, which is the C-term carboxamide version of **5a**. While the observed high proteolytic stability of cyclic peptides **5**–**8**, especially **6**–**8**, is consistent with the notion that cyclic peptides also tend to be more proteolytically stable than linear peptides [27], this also suggests that the cyclic peptide-based sirtuin substrates may also be useful in settings where native proteases/peptidases are present, such as cell lysates and intracellular compartments.

## 3. Experimental Section

### 3.1. General

The following materials were obtained from commercial sources for the compound synthesis and purification, and were used as received. Sigma-Aldrich China (Shanghai, China): *N*-methylmorpholine (NMM), trifluoroacetic acid (TFA), *N*,*N*-dimethylformamide (DMF), hydrazine monohydrate, Rink amide resin; TCI Shanghai (Shanghai, China): suberic acid, succinic acid, myristic acid; Alfa Aesar China (Shanghai, China): phenol, thioanisole, ethanedithiol, *N*-hydroxybenzotriazole (HOBt), 2-(1H-benzotriazole-1-yl)-1,1,3,3-tetramethylaminium hexafluorophosphate (HBTU); Honeywell China (Shanghai, China): acetonitrile, dichloromethane (DCM); Sinopharm Chemical Reagent Co., Ltd. (Shanghai, China): piperidine, diethyl ether, acetic anhydride.

The N^α^-Fmoc-protected amino acids were purchased from Sigma-Aldrich China, Alfa Aesar China, TCI Shanghai, or GL Biochem (Shanghai) Ltd. (Shanghai, China).

The high-resolution mass spectrometry (HRMS) was performed on an AB 5600+ Q TOF high-resolution mass spectrometer (AB Sciex LLC, Framingham, MA, USA) at the Pharmacy School of Fudan University. RP-HPLC analyses were performed on a Shimadzu LC-20AT station (Nakagyo-ku, Kyoto, Japan).

The following materials were obtained from commercial sources for the in vitro sirtuin deacylation activity assay and the pronase digestion assay, and were used as received. Sigma-Aldrich China: the active human recombinant His_6_-SIRT1, Trizma, Hepes, β-NAD^+^, a 1.0 M solution of MgCl_2_ (molecular-biology grade), the pronase from *Streptomyces griseus*; Cayman Chemical (Ann Arbor, MI, USA): the active human recombinant GST-SIRT1, the active human recombinant His_6_-SIRT2, the active human recombinant His_6_-SIRT3; TCI Shanghai: DL-dithiothreitol (DTT); Alfa Aesar China: NaCl, KCl.

### 3.2. Compound Preparation

The following describe the preparation of compounds **5**–**8**, all starting from the Rink amide resin. The purified compounds **5**–**8** were obtained with overall yields of ~50–70%.

#### 3.2.1. Preparation of Compounds **5** and **6** (Scheme 1)

A side-chain fully protected and N-terminus acetylated linear pentapeptide (i.e., CH_3_CONH-Lys(Mtt)-Lys(Boc)-(N^ε^-acetyl-Lysine)-Lys(Mtt)-Met) was initially assembled on the Rink amide resin (0.1 mmol) based on the Fmoc chemistry-based manual SPPS as follows. For each amino acid coupling, 4 equivalents of a N^α^-Fmoc-protected amino acid, 3.8 equivalents of the coupling reagent HBTU, and 3.8 equivalents of the additive HOBt were used in the presence of 0.4 M NMM/DMF (2 mL), and the coupling reaction was allowed to proceed at room temperature for 1 h; A 20% (*v*/*v*) piperidine/DMF solution (5 mL) was used for Fmoc removal (10 min × 2). Subsequently, the side chain Mtt protecting group on Lys(Mtt) was selectively removed with a 1% (*v*/*v*) TFA/DMF solution (5 mL) (2 min × 9). The obtained peptidyl-resin with two exposed free amino groups was then divided into two equal portions and used for the synthesis of compounds **5** and **6** as follows. For the synthesis of **5**, it was reacted with Fmoc-Gly-OH under amino acid coupling condition, followed by (i) Fmoc removal with 20% (*v*/*v*) piperidine/DMF (2.5 mL) (10 min × 2) and (ii) the subsequent reaction between the two newly exposed free amino groups on resin with suberic acid (0.1 mmol, 2 equivalents) in the presence of HBTU (0.19 mmol, 3.8 equivalents) and 0.4 M NMM/DMF (1 mL) at room temperature for 1 h. For the synthesis of **6**, it was reacted with succinic acid (0.1 mmol, 2 equivalents) in the presence of HBTU (0.19 mmol, 3.8 equivalents) and 0.4 M NMM/DMF (1 mL) at room temperature for 1 h. The peptidyl resin obtained from the above-described reaction with a di-acid (suberic acid or succinic acid) was then treated with a TFA-containing solution (90% (*v*/*v*) TFA, 5% (*v*/*v*) DCM, 5% *(v*/*v*) ddH_2_O) (2.9 mL) at room temperature for 4 h to cleave **5** or **6** off the resin. Specifically, a cleavage mixture was filtered and the volatiles in the filtrate were removed with a stream of nitrogen gas in a well-ventilated fuming hood, and from the residue the crude **5** or **6** was precipitated out and washed with cold diethyl ether (2 mL and 2 × 2 mL, respectively). The obtained crude **5** and **6** were then purified by RP-HPLC on a semi-preparative C18 column (1 × 25 cm, 5 µm). The column was eluted with a gradient of ddH_2_O containing 0.05% (*v*/*v*) TFA and acetonitrile containing 0.05% (*v*/*v*) TFA at 4.5 mL/min and was monitored at 214 nm. The pooled desired HPLC fractions were concentrated with a rotary evaporator to remove acetonitrile and the remaining aqueous solutions were lyophilized to afford the purified **5** and **6** as puffy white solids. The exact masses of the purified **5** and **6** were confirmed by HRMS analysis (Table 1). The purities of the purified **5** and **6** were each >95% based on a RP-HPLC analysis on an analytical C18 column (0.46 × 25 cm, 5 μm).

#### 3.2.2. Preparation of Compound **7** (Scheme 2)

A side-chain fully protected and N-terminus acetylated linear pentapeptide (i.e., CH_3_CONH-Lys(Mtt)-Arg(Pbf)-Lys(ivDde)-Lys(Mtt)-Thr(tBu)) was initially assembled on the Rink amide resin (0.05 mmol) based on the Fmoc chemistry-based manual SPPS, as described above. The two Mtt protecting groups on Lys(Mtt) were then selectively removed with 1% (*v*/*v*) TFA/DMF (2.5 mL) (2 min × 9). The resulting peptidyl-resin with two exposed free amino groups was then reacted with suberic acid (0.1 mmol, 2 equivalents) in the presence of HBTU (0.19 mmol, 3.8 equivalents) and 0.4 M NMM/DMF (1 mL) at room temperature for 1 h. The obtained peptidyl-resin was subsequently treated with a 2% (*v*/*v*) solution of hydrazine (NH_2_NH_2_) in DMF (5 mL) to selectively remove the ivDde protecting group on Lys(ivDde) (2 × 1 h at room temperature). The newly exposed free amino group was then reacted with myristic acid (0.2 mmol, 4 equivalents), HBTU (0.19 mmol, 3.8 equivalents), and HOBt (0.19 mmol, 3.8 equivalents) in the presence of 0.4 M NMM/DMF (1 mL). The peptidyl-resin thus obtained was subsequently treated with Reagent K (83.6% (*v*/*v*) TFA, 5.9% (*v*/*v*) phenol, 4.2% (*v*/*v*) ddH_2_O, 4.2% (*v*/*v*) thioanisole, 2.1% (*v*/*v*) ethanedithiol) (2.9 mL) at room temperature for 4 h to cleave **7** off the resin. Specifically, after a cleavage mixture was filtered and the volatiles in the filtrate were removed with a stream of nitrogen gas in a well-ventilated fuming hood, to the residue was added cold diethyl ether (2 mL and 2 × 2 mL, respectively) to precipitate out and wash the crude **7** which was then purified by RP-HPLC on a semi-preparative C18 column (1 × 25 cm, 5 µm), as described above, affording the purified **7** as a puffy white solid. The exact mass of the purified **7** was confirmed by HRMS analysis (Table 1). The purity of the purified **7** was >95% based on a RP-HPLC analysis on an analytical C18 column (0.46 × 25 cm, 5 μm).

#### 3.2.3. Preparation of Compound **8** (Scheme 3)

Compound **8** was synthesized according to Scheme 3 in the same manner as the above-described synthesis of compound **5**. The crude **8** was also purified with semi-preparative RP-HPLC and the exact mass of the purified **8** was also confirmed by HRMS analysis (Table 1). The purified **8** was also >95% pure based on RP-HPLC analysis.

### 3.3. Kinetic Parameter Determination for ***5**–**8***’s Substrate Activities with SIRT1/2/3

A sirtuin deacylation assay solution contained the following: 50 mM Hepes (pH 8.0), 137 mM NaCl, 2.7 mM KCl, 1 mM MgCl_2_, 1 mM DTT, β-NAD^+^ (~5.6 × *K*_M_: 0.5 mM for the SIRT1 and SIRT2 assays, 3.5 mM for the SIRT3 assay), one test compound (**5**, **6**, **7**, or **8**) with varied concentrations including 0, and a sirtuin (His_6_-SIRT1 or GST-SIRT1, 243 nM; His_6_-SIRT2, 297 nM; or His_6_-SIRT3, 739 nM). An enzymatic reaction was initiated by the addition of a sirtuin at 37 °C and was incubated at 37 °C for 5 min (for the SIRT1 assay) or 2 min (for the SIRT2 and SIRT3 assays) until quenched with the following stop solution: 100 mM HCl and 0.16 M acetic acid. The quenched assay solutions were directly injected into a C18 column (0.46 × 25 cm, 5 μm), and the column was eluted with a gradient of ddH_2_O containing 0.05% *(v*/*v)* TFA and acetonitrile containing 0.05% (*v*/*v*) TFA at 1 mL/min and was monitored at 214 nm. Turnover of the limiting substrate was kept at <10%. Stock solutions of the test compounds were all prepared in ddH_2_O. The integrated product and substrate HPLC peak areas were then used to determine the initial velocities for the sirtuin-catalyzed deacylation reaction at different concentrations of a test compound (**5**, **6**, **7**, or **8**). The initial velocity-concentration data were analyzed with KaleidaGraph^®^ (Synergy Software, Reading, PA, USA) and the data were fitted to the Michaelis-Menten equation to obtain the kinetic parameters *k_cat_* and *K_M_*, as recorded in Table 2 and Table 3. Of note, non-enzymatic control runs did not yield deacylation product even when the highest concentration of a test compound (i.e., **5**, **6**, **7**, or **8**) was used.

### 3.4. Pronase Digestion Assay

This assay was performed according to the procedure described previously by our laboratory [35]. A solution of a test compound (**5**, **6**, **7**, or H_2_N-HK-(N^ε^-acetyl-lysine)-LM-COOH) in ddH_2_O (160 µM) was mixed with equal volume of a pronase solution in 100 mM Tris HCl (pH 7.3) (8 ng/µL); the resulting well-mixed solution was then incubated at 37 °C until quenched with a 1.0 M solution of acetic acid in ddH_2_O at 0, 7.5, 15, 30, and 60 min (for **5**, **6**, and **7**) or 0, 1.5, 3, and 6 min (for H_2_N-HK-(N^ε^-acetyl-lysine)-LM-COOH). At each time point, 20 µL of a pronase digestion solution was taken and treated with 40 µL of the 1.0 M acetic acid aqueous solution; and the whole mixture was vortexed vigorously, centrifuged, and the supernatant was injected into a RP-HPLC analytical C18 column (0.46 × 25 cm, 5 μm). The column was eluted with a gradient of ddH_2_O containing 0.05% (*v*/*v*) TFA and acetonitrile containing 0.05% (*v*/*v*) TFA at 1 mL/min with ultraviolet monitoring at 214 nm. The HPLC peak areas for a given test compound at different time points were used to estimate the percentage remaining for this test compound versus digestion time. The graph of the percentage remaining versus digestion time was used to compare the proteolytic stability of different test compounds, as shown in Figure 4.

## 4. Conclusions

In the current study, we found that several cyclic peptides harboring N^ε^-acetyl-lysine or N^ε^-myristoyl-lysine behaved as superior in vitro SIRT1 or SIRT3 substrates (as judged by the *k*_cat_/*K*_M_ ratios) compared to the best linear hexapeptide-based in vitro SIRT1 or SIRT3 substrates reported in the current literature. Moreover, these cyclic peptides were also found to be proteolytically much more stable than a linear pentapeptide control. These cyclic peptide-based substrates may be also useful in in vitro screening platforms for sirtuin chemical modulator discovery; if cell permeable, they may also be used to assess intracellular sirtuin deacylation activities when combined with the use of the potent/selective/cell permeable sirtuin deacylation inhibitors.

## Figures and Tables

**Table 1:** The high-resolution mass spectrometry (HRMS) analysis of the purified compounds **5**–**8** ^a^.

Compound	Ionic Formula	Calculated *m*/*z*	Observed *m*/*z*
**5**	[C_45_H_81_N_12_O_11_S]^+^	997.5863	997.5856
**6**	[C_37_H_67_N_10_O_9_S]^+^	827.4808	827.4778
**7**	[C_52_H_97_N_12_O_10_]^+^	1049.7445	1049.7449
**8**	[C_40_H_71_N_11_O_10_Na]^+^	888.5278	888.5282

^a^ All compounds were measured with electrospray ionization (positive ion mode).

**Table 2:** The kinetic parameters for compounds **5**/**6**’s substrate activities with SIRT1/2/3 ^a^.

Compound	5			6		
Sirtuin	*K*_M_ (μM)	*k*_cat_ (10^−3^, s^−1^)	*k*_cat_/*K*_M_ (M^−1^·s^−1^)	*K*_M_ (μM)	*k*_cat_ (10^−3^, s^−1^)	*k*_cat_/*K*_M_ (M^−1^·s^−1^)
SIRT1	22.2 ± 1.8	35.6 ± 5.1	(1.6 ± 0.1) × 10^3^	41.3 ± 1.83	25.9 ± 2.3	(0.63 ± 0.03) × 10^3^
SIRT2	11.6 ± 2.2	36.0 ± 4.9	(3.2 ± 1.0) × 10^3^	49.7 ± 7.78	138.9 ± 26.7	(2.79 ± 0.1) × 10^3^
SIRT3	4.5 ± 0.9	15.8 ± 1.4	(3.6 ± 0.4) × 10^3^	34.9 ± 4.04	155.3 ± 16.6	(4.51 ± 1.0) × 10^3^

^a^ See Experimental Section for assay details.

**Table 3:** The kinetic parameters for compounds **7**/**8**’s substrate activities with SIRT1/2/3 ^a^.

Compound	7			8		
Sirtuin	*K*_M_ (μM)	*k*_cat_ (10^−3^, s^−1^)	*k*_cat_/*K*_M_ (M^−1^·s^−1^)	*K*_M_ (μM)	*k*_cat_ (10^−3^, s^−1^)	*k*_cat_/*K*_M_ (M^−1^·s^−1^)
SIRT1	2.85 ± 0.13	5.8 ± 0.1	(2.04 ± 0.06) × 10^3^	42.2 ± 17.5	90.2 ± 39.4	(2.2 ± 0.05) × 10^3^
SIRT2	37.6 ± 14.4	90.9 ± 2.62	(2.6 ± 0.93) × 10^3^	43.1 ± 16.0	414.0 ± 25.0	(10.4 ± 4.4) × 10^3^
SIRT3	4.2 ± 0.38	60.0 ± 2.8	(14.3 ± 0.66) × 10^3^	99.9 ± 48.7	221.0 ± 32.0	(2.4 ± 0.9) × 10^3^

^a^ See Experimental Section for assay details.
